# Development of a spatial sampling protocol using GIS to measure health disparities in Bobo-Dioulasso, Burkina Faso, a medium-sized African city

**DOI:** 10.1186/s12942-017-0087-7

**Published:** 2017-04-18

**Authors:** Daouda Kassié, Anna Roudot, Nadine Dessay, Jean-Luc Piermay, Gérard Salem, Florence Fournet

**Affiliations:** 1 Université Paris Ouest Nanterre La Défense, 200 Avenue de la République, 92000 Nanterre, France; 2 CIRAD, ASTRE, CIRAD TA C-22/E, Campus International de Baillarguet, 34398 Montpellier Cedex 5, France; 3 ESPACE DEV, Institut de Recherche pour le Développement, Maison de la Télédetection, 500 rue Jean-François Breton, 34093 Montpellier Cedex 5, France; 4 Université De Strasbourg, 4 Rue Blaise Pascal, 67081 Strasbourg, France; 5 CEPED, Institut de Recherche pour le Développement, 19 Rue Jacob, 75006 Paris, France; 6 MIVEGEC, Institut de Recherche pour le Développement, 911, Avenue Agropolis, BP 64501, 34394 Montpellier Cedex 5, France; 7 Institut de Recherche en Sciences de la Santé, 01 BP 545, Bobo-Dioulasso, Burkina Faso

**Keywords:** Health disparities, Spatial sampling, Typology, Medium-sized city, Bobo-Dioulasso

## Abstract

**Background:**

Many cities in developing countries experience an unplanned and rapid growth. Several studies have shown that the irregular urbanization and equipment of cities produce different health risks and uneven exposure to specific diseases. Consequently, health surveys within cities should be carried out at the micro-local scale and sampling methods should try to capture this urban diversity.

**Methods:**

This article describes the methodology used to develop a multi-stage sampling protocol to select a population for a demographic survey that investigates health disparities in the medium-sized city of Bobo-Dioulasso, Burkina Faso. It is based on the characterization of Bobo-Dioulasso city typology by taking into account the city heterogeneity, as determined by analysis of the built environment and of the distribution of urban infrastructures, such as healthcare structures or even water fountains, by photo-interpretation of aerial photographs and satellite images. Principal component analysis and hierarchical ascendant classification were then used to generate the city typology.

**Results:**

Five groups of spaces with specific profiles were identified according to a set of variables which could be considered as proxy indicators of health status. Within these five groups, four sub-spaces were randomly selected for the study. We were then able to survey 1045 households in all the selected sub-spaces. The pertinence of this approach is discussed regarding to classical sampling as random walk method for example.

**Conclusion:**

This urban space typology allowed to select a population living in areas representative of the uneven urbanization process, and to characterize its health status in regards to several indicators (nutritional status, communicable and non-communicable diseases, and anaemia). Although this method should be validated and compared with more established methods, it appears as an alternative in developing countries where geographic and population data are scarce.

## Background

Urbanization is a phenomenon that modifies the environment and living conditions on all continents. Since 2007, more than half of the world population live in cities and this percentage is expected to increase to almost 70% in 2050 [1], and to 50% for West Africa from now to 2030. Many cities in developing countries experience an unplanned growth that, as a result, exposes the populations to numerous environmental risks with complex and still poorly known health consequences.

Cities are dense (concentration of populations), open (high mobility due to, for instance, migrations from and to rural environments) and heterogeneous environments. This heterogeneity is not linked only to the uneven distribution of infrastructures [2, 3]. It is also caused by the urbanization process on its own. Indeed, a city is not built in the same way if it develops in a plain or in the middle of mountains, or if it is traversed by low-lying grounds or close to the seaside, or if its growth is controlled or not. Several studies have shown that the irregular urbanization and patchy infrastructure of cities have many consequences on health [3–5], by producing different health risks and uneven risk of exposure to specific diseases [6]. Many of these studies were conducted in big cities, although urbanization occurs more and more in medium-sized cities [7]. These places have the disadvantages of cities (unplanned growth, pollution) and also of rural areas (under-equipment) and their consequences on health have been not fully investigated [8].

The study of urban health disparities is complex for different reasons. First of all, the health status of a population is influenced not only by individual factors, but also by a multitude of genetic, social, demographic, cultural, physical, economic and also political determinants that interact with each other [9]. Therefore, due to the urban heterogeneity, it is clear that a prevalence given for the whole city will mask intra-urban differences that are not without consequences on the population quality of life [2, 4]. For instance, infant deaths are less frequent in Nairobi than in rural areas of Kenya; however, this urban mean value hides substantial variations within the city. Indeed, the mortality rate of deprived areas is much higher than that of the whole city [1].

Consequently, the need of working at the micro-local scale becomes obvious [10]. However, a major issue is to know how to capture this urban diversity and how to analyse health in the light of such diversity. The quantitative and qualitative characterization of urbanization remains a black box, particularly in low-income countries. Although in many cities, the centre is distinguished from the peripheries, this dichotomy is not always present. Moreover, it cannot be reduced to a gradient that translates a progressively stronger urbanization from the periphery towards the centre, for example. In the cities of developing countries, often a high-quality house stands alongside a very precarious house. According to Grafmeyer [11], a city is at the same time a territory and also a population, a framework collective life, a collection of physical objects and a cluster of relationships. Indeed, although a city is the results of a long-term construction, it cannot be dissociated from those that are building it. Therefore, the urban space should be considered as the support, product and subject of social relationships [4].

In developing country settings, old censuses as well as lack of health surveillance and geographic data limit survey design options. In these situations, approaches require to propose new sampling frames. Expanded programme on immunization (EPI) method may be a solution but is often difficult to apply in urban settings [12]. Alternatives based on adaptation of EPI method [13], or on purposeful selection of clusters guided by knowledge of the spatial arrangement of key population characteristics [14] could be proposed. But the challenge remains to develop a method allowing sampling of specific sub-spaces which illustrate the urban diversity, and of populations who participate to this diversity while providing approaches applicable to other contexts.

To determine the effect of the production of an urban space on the health in a medium-sized city, we developed a multi-stage sampling method adapted to our objective. Our aim was to investigate the health status of Bobo-Dioulasso (Burkina Faso) populations at the intra-city scale, in view of presenting concrete proposals to the municipal authorities, healthcare and urban planning policy-makers. The chosen methodological approach combined the use of different tools with qualitative approaches that allowed the fine characterization of the urban space of Bobo-Dioulasso relative to health questions. The obtained city typology was used to select study areas that are representative of different urbanization modes, assuming that population health status would be different. The health status was described based on different indicators of communicable (malaria and dengue) and non-communicable diseases (blood hypertension and diabetes), nutritional status and anaemia. This choice was justified by the growing evidence of a double burden of diseases in cities of developing countries [15].

The different steps of this approach are presented as well as the results in terms of choice of sub-spaces and surveyed populations.

## Methods

### Study site

**Fig. 1:**
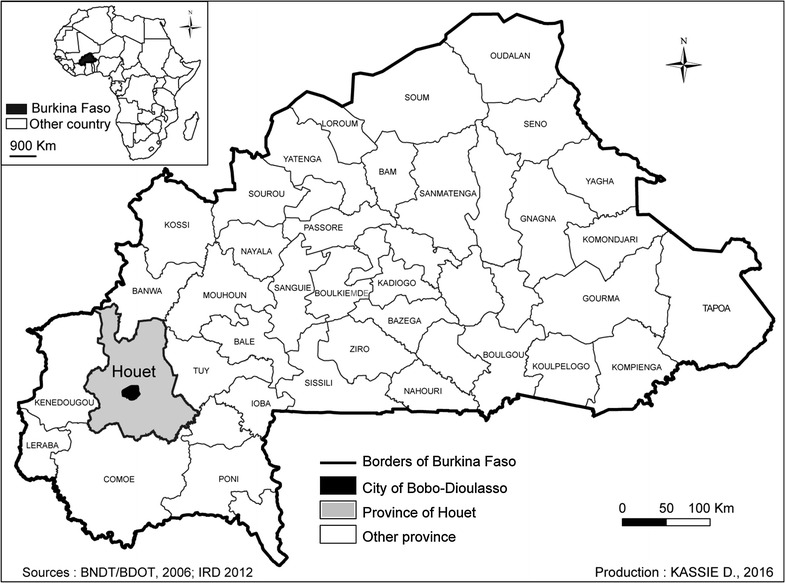
Location of the study area

The city of Bobo-Dioulasso is in the West of Burkina Faso, in the middle of Houet province of which it is the main city (Fig. 1).

**Fig. 2:**
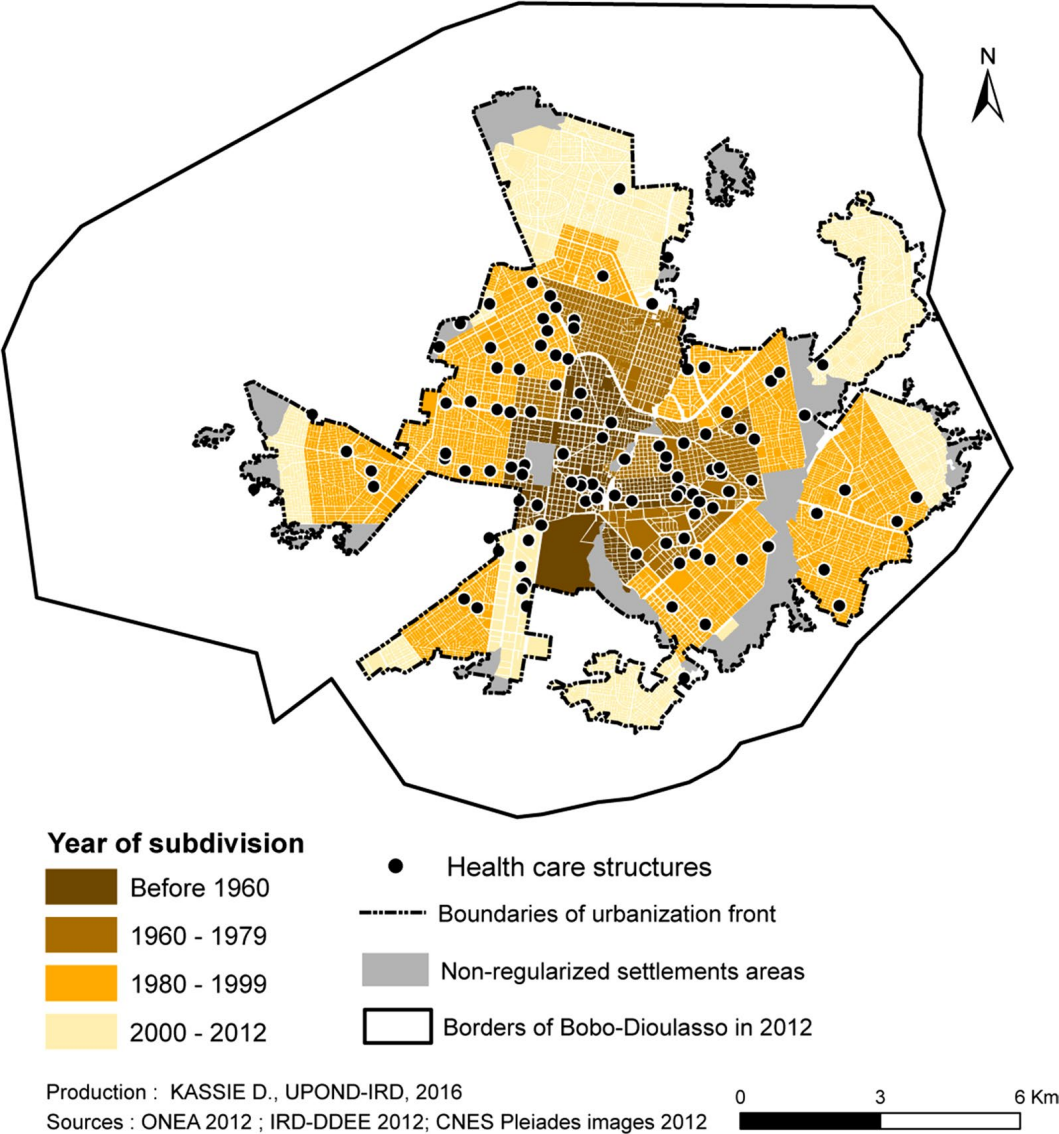
Spatial distribution of healthcare structures in Bobo-Dioulasso from 1960 to 2012 in relation with the subdivision

**Fig. 3:**
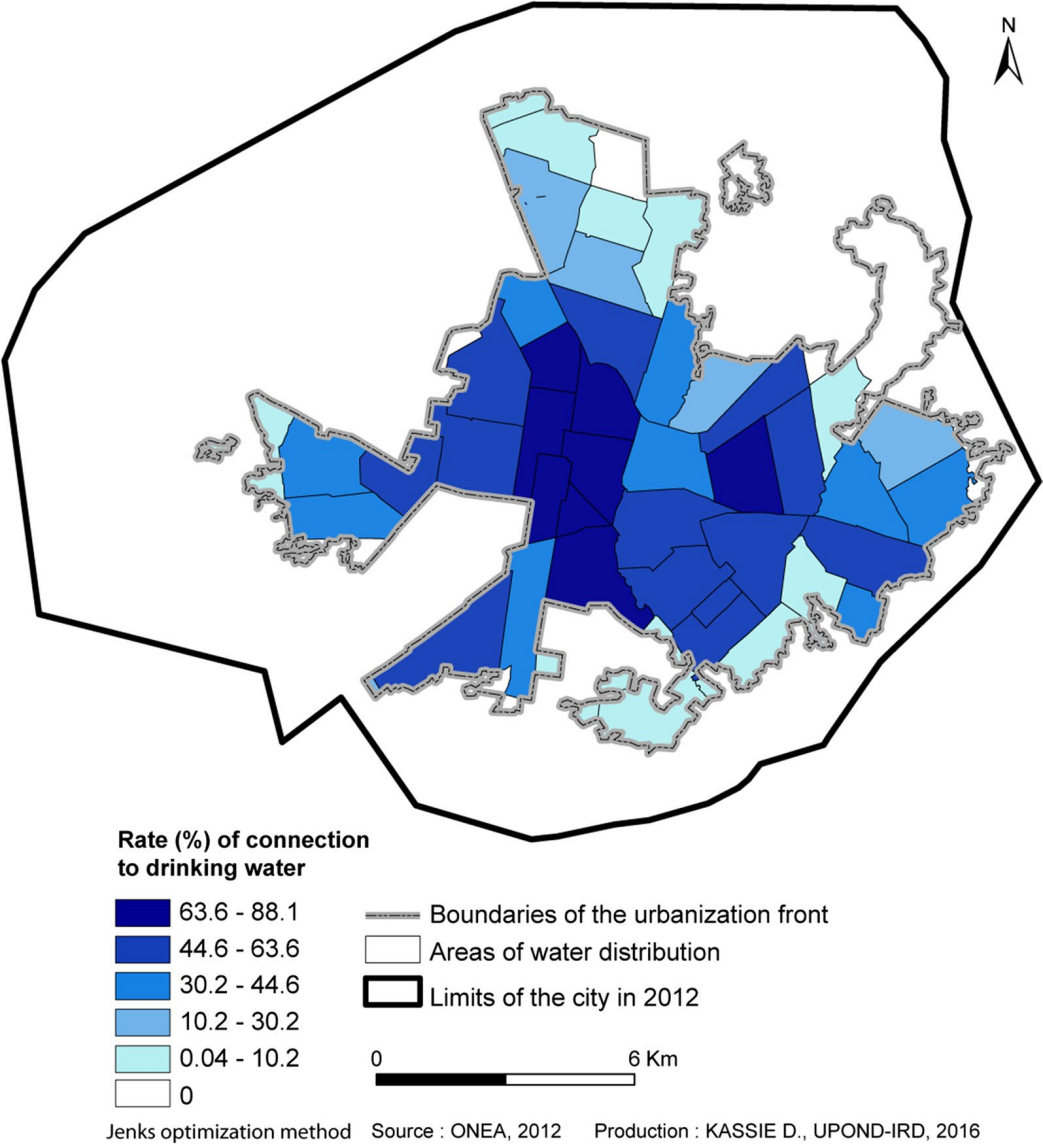
Rate of connection to drinking water in the serviced areas defined by ONEA in Bobo-Dioulasso in 2012

Bobo-Dioulasso has been showing relatively important and constant growth rates (4.7% per year, on average) since its discovery by colonialists, with the exception of some key periods, particularly 1985–1996. During this period, all the efforts were focused on Ouagadougou, which was the showcase of the Sankarist urban policy, and Bobo-Dioulasso was left outside the fast evolutionary dynamics [16, 17]. This expansion was not without consequences on the urban planning. An irregular distribution of healthcare services in space and time in Bobo-Dioulasso was observed. The installation of healthcare structures did not always follow the major phases of urban growth due to the different policies started by the State and international institutions. Thus, the number of healthcare structures rose from four before 1960 to 52 in 2012, without an even coverage of the entire urban space [18] (Fig. 2). Similarly, differences concerning the access to drinking water were observed, with inexistent rates of connection in non-regularized settlements and variable rates in regularized areas (Fig. 3).

### Data sources

The literature on the history, social organization and urbanization of Bobo-Dioulasso was reviewed. Aerial photographs from 1952 to 1987 (Institut Géographique National, in English French National Geographic Institute), and aerial photographs of 1994 (Institut Géographique du Burkina, in English: Burkina Faso Geographical Institute) were used to retrace the city growth in time and space. These photographs were georeferenced and then mosaics were generated for each year.

The satellite images used for this study corresponded to: (1) two SPOT 5 images of 2004 and 2007 at high definition acquired in multi-spectral (M) at 10 m and panchromatic (P) mode from the ISIS programme of CNES (the French space agency). For 2004, the spatial resolution for the P mode image was 5 m and for 2007 it was 2.5 m (generated from two images at 5 m acquired simultaneously); (2) one Pléiades satellite image at very high resolution of 2012 also from CNES. Its resolution was 2.8 m for the M mode and 0.7 m re-sampled to 0.5 m for the P mode. The images obtained with the optic system SPOT 5 present the advantage of covering a large geographical area (60 km). The Pléiades images cover a reduced area (20 km), but allow the detection of objects smaller than 1 m.

The 2012 cadastral map of Bobo-Dioulasso was obtained from the National Water and Sanitation Office (Office National de l’Eau et de l’Assainissement, ONEA) of the city.

### Methodological approach

The methodological approach was developed to meet different objectives: (a) to establish the urban growth and the displacement of the urbanization front by visual interpretation of photographs for the following years: 1952, 1958, 1964, 1980, 1987, 1994 and of scenes for the following years: 2004, 2007 and 2012; (b) to divide the city in homogeneous spatial units based on the size and the organization of the built environment, the configuration of the road network, and the presence of vegetation by visual interpretation of the scene of 2012; (c) to extract the city’s fabric, the vegetation and the road network from the satellite image of 2012 to produce a spatial database that should facilitate the analysis of health disparities (Table 1); (d) to produce a typology of the city based on spatial analysis (principal component analysis and hierarchical ascendant classification).

**Table 1:** Overview of aerial photographs and remote sensing datasets

Providers	Datasets	Date	Spatial resolution	Type of analysis
IGN France	5 Aerial photographs	1952	1/15,000	Analysis of the urban growth (photo-interpretation)
	9 Aerial photographs	1958	1/15,000	
	6 Aerial photographs	1964	1/26,000	
	5 Aerial photographs	1980	1/20,000	
	9 Aerial photographs	1987	1/20,000	
IGB	11 Aerial photographs	1994	1/20,000	
CNES	1 SPOT panchromatic image	2004	5 m	
	1 Multispectral Image	2004	10 m	
	1 SPOT panchromatic image	2007	2.5 m	
	1 Multispectral image	2007	10 m	
CNES	2 Pléiades high resolution panchromatic images	2012	0.5 m	Analysis of the urban growth and identification of the AHU (photo-interpretation, supervised classification of the urban morphology)
	2 Pléiades high resolution Multispectral images	2012	2 m	

The definition of spatial units inside which health status was surveyed allowed to throw of classical administrative division like districts or census blocks, which did not necessary ensure to evaluate how neighbourhoods may affect individual or population health [19, 20].

#### Urban growth and identification of AHU by visual photo-interpretation

From 1952 to 2012, the mosaics of aerial photographs and remote sensing images were visually interpreted to delimit the urbanization front and to determine the growth of the urban space. The urban fabric morphology was then analysed to obtain fine and precise information on the shapes of subsets within the city. This analysis took into account simultaneously the built space, the space covered by vegetation and the road network [21–24]. Hence, the space will appear in the shape of urban subsets that are characterized by their coherence and unity. This was realized with the Pléiades images of 2012 by visual interpretation and led to delineate morphologically similar spaces called areas of homogeneous units (AHU). These spatial units were delimitated around urban blocks comprising a variable number of plots.

This approach gave a structured and synthetic vision of the urban fabric by putting it in the global context. It allowed the categorization of a city in different urban fabrics that could be charted, thus producing a good image of what could be observed in the field. Therefore, this segmentation allowed studying the space continuity and the areas where it breaks up.

#### Supervised classification of the urban morphology at very high spatial resolution

The aims of this classification were: (a) to move from the photo-interpretation towards a generic method, (b) to put in place a stratified sampling method in order to optimize the use of classes that have a direct or potential link with health as sampling criteria for the areas to be surveyed.

The supervised classification by maximum similarity was performed starting from the Pléiades image of 2012. Two classes were added to the already identified thematic classes: water collections (water puddles, river, ponds) and bare soil (sand, laterite). Asphalted roads were differentiated within the road network class.

Different indexes were calculated: Normalized Vegetation Index, Humidity Index and Brightness Index. The confusions identified based on the confusion matrix were corrected after integration of the results in a geographic information system (GIS). This is the class “clouds” when confused with the class “urban” and the class “building” when attached to the wrong urban component. These corrections were implemented manually.

The results of these post-treatments allowed calculating the density of vegetation and of built surfaces in each AHU and then using these density values for spatial analyses, specifically the principal component analyses (PCA) and hierarchical ascendant classification (HAC).

Field surveys were also carried out to localize the urban infrastructures (healthcare structures, schools, water fountains, traffic lights, markets, coach stations) and to validate the satellite image-based analyses. They were completed with surveys in the healthcare structures to collect additional information on the structure type and opening year.

Finally, these data were completed by surveys based on the statements of responsible people at the town hall, ONEA, waste management service, associations, media and traditional and religious rulers.

#### Generation of a town typology

Its objective was to select in a robust manner the urban sub-spaces in which the population will be surveyed. A sampling method that combined PCA and HAC was applied on the AHU identified at the precedent stage to generate the city typology. This allows improving the sampling quality compared with the simple random sampling technique^1^ by taking into account simple, objective and measurable criteria [23], such as the built surfaces or those covered by vegetation. A similar method was used in Dakar to analyse socio-economic disparities [25].

PCA generates new artificial variables and graphic representations that allow visualizing the relationships between variables or between individuals (spatial units), as well as the possible existence of relationships between groups of individuals or between groups of variables. It detects and reduces the number of correlated variables to be used for HAC. HAC objective is to find, in several steps, the closest classes and then to merge them till only one class remains [26]. Several variables that discriminate the urban space were retained to identify via PCA and HAC the sub-spaces that represent diversified urbanization processes where the health status may vary. The chosen variables measured health vulnerability (building density, level of infrastructures, risk of flooding, age of the district) or the access to urbanization (access to healthcare structures and to drinking water). They could be expressed by a mean (e.g., mean building density within the AHU), by a percentage (e.g., portion of the AHU at risk of flooding) or even by a standard deviation (e.g., standard deviation of the rate of connection to the drinking water network within the AHU).

These analyses generated a typology of Bobo-Dioulasso that could be used to select urban sub-spaces for health surveys. Within each class identified by the HAC, a sub-space was randomly selected. Only one sub-space was selected in each class as the objective was to compare the health disparities between the different classes, and not between different AHU belonging to a same class.

#### Spatial and demographic sampling for the health survey

After the identification of the four AHU to be surveyed, several spatial random sampling without replacement allowed selecting plots within each AHU using the cadastral map of 2012. This method ensures the comparability of the data collected in the different AHU. The Pléiades image and the cadastral map of 2012 were used to identify and eliminate from the sampling, uninhabited plots dedicated to administrative or commercial usage. In regular areas, plots were randomly selected using the ‘Sampling Design Tool’ of ArcGIS 10. For non-regular areas, without delimited plots, houses were digitalized by referring to the roof and their geographical coordinates were integrated in the GIS for the sampling.

Ripley’s K-function which is typically used to compare a given point distribution with a random distribution, was used to test the spatial distribution of the selected households in each AHU [18]. The point distribution is tested against the null hypothesis that the points are distributed randomly and independently. We used a common transformation of the K-Function, often referred to as L(d) which is implemented in ArcGIS. When the observed K value is larger than the expected K value for a particular distance, the distribution is more clustered than a random distribution at that distance (scale of analysis). When the observed K value is smaller than the expected K, the distribution is more dispersed than a random distribution at that distance.

The geographical coordinates of the randomly selected plots or digitalized houses were integrated in Garmin eTrex 10 handheld GPS units. Surveyors had to find these concessions by using the procedure ‘Go to’ of the GPS unit and with the help of maps on which these plots were shown. Each survey sub-space was subdivided in three parts and each part was attributed to a surveyor who had to cover it completely. This technique avoided having all the selected households in a single part of the study area.

**Fig. 4:**
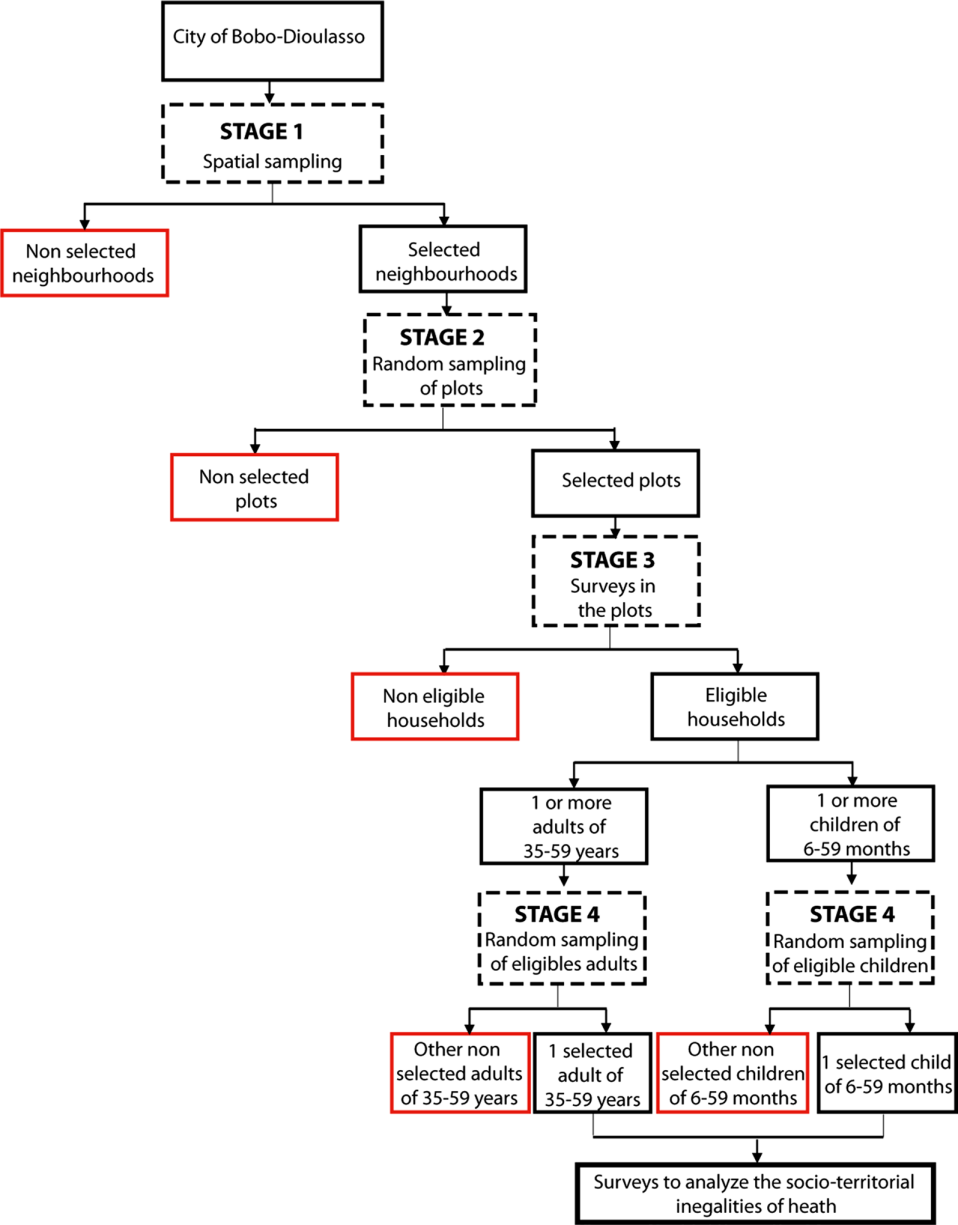
Schematic of the surveys carried out in Bobo-Dioulasso in 2013

To be easily identified by the populations, each surveyor had a badge and a work kit (GPS unit, map with the randomly selected points, forms for data collection, etc.). As one or more households may live inside a same plot or a same house in non-regularized areas, the surveyor counted the number of households and identified those eligible for the survey before selecting one by random sampling. A household was eligible if it included at least one eligible child (6–59 months of age) and one eligible adult (35–59 years of age) (Fig. 4).

First, the surveyor needed to collect the household head’s authorization for the household participation in the survey after eligibility verification. In the case of absence or doubts on the age of the people to be surveyed, the surveyor took an appointment. After three unsuccessful appointments, the household was abandoned and replaced by another household in the random sampling list for that sub-space.

**Fig. 5:**
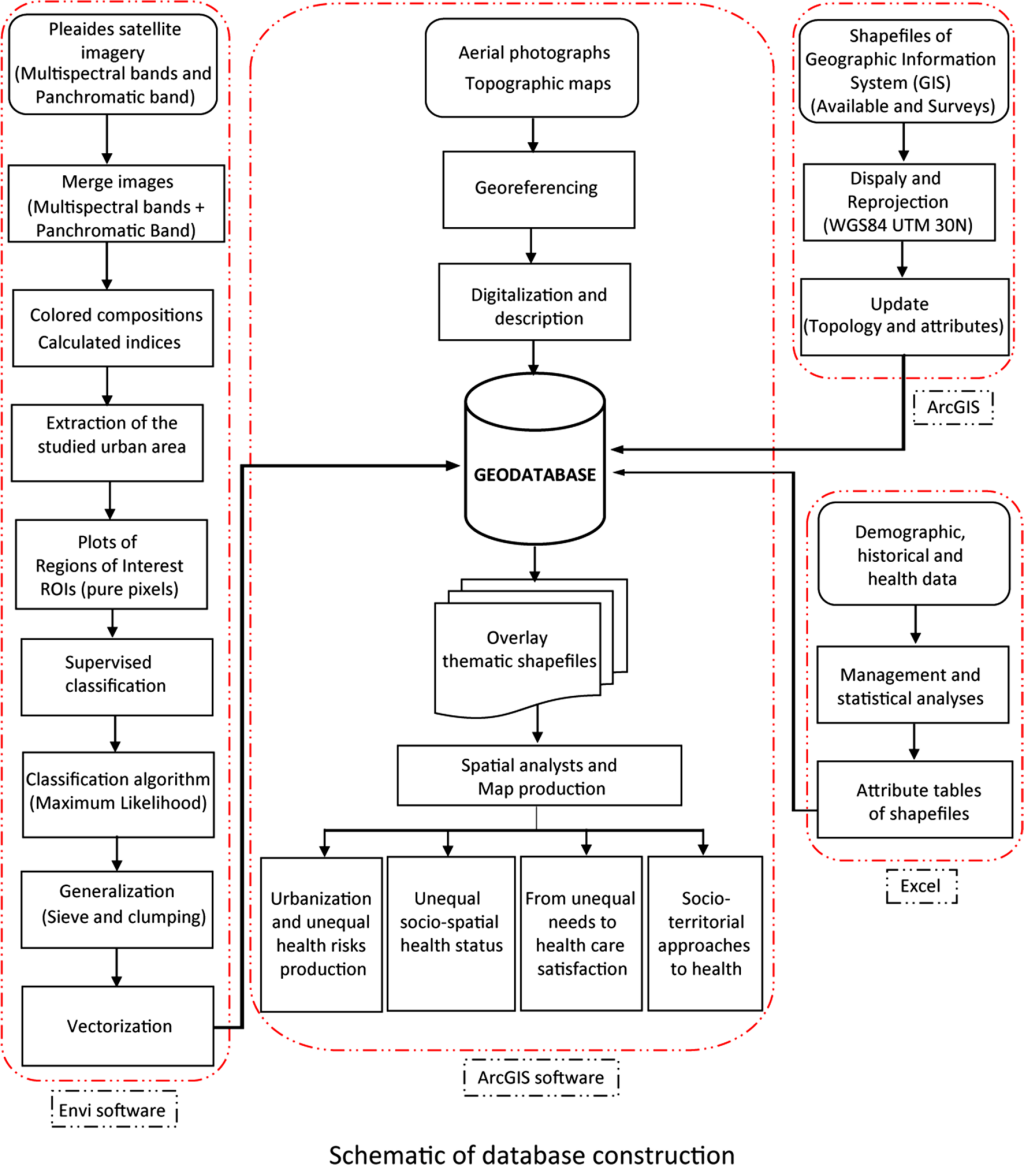
Schematic of the database construction methodology

All collected and analysed geographical data^2^ (aerial photographs, satellite images, geographical coordinates of urban infrastructures) were integrated in a geodatabase with a map projection WGS84 UTM 30 N (Fig. 5).

In order to observe significant differences between AHU at the 5% threshold with a precision of 2.6% based on a prevalence of 5% (which was the smallest expected prevalence for diabetes in adults), a sample size of 250 adults and 250 children (and then 250 households) was calculated. The same number of adults and children were chosen in each AHU to allow the comparability between AHU by multivariate analysis. This method permitted to determine if the same risk factors were associated to the same health indicator in each AHU.

## Results

**Fig. 6:**
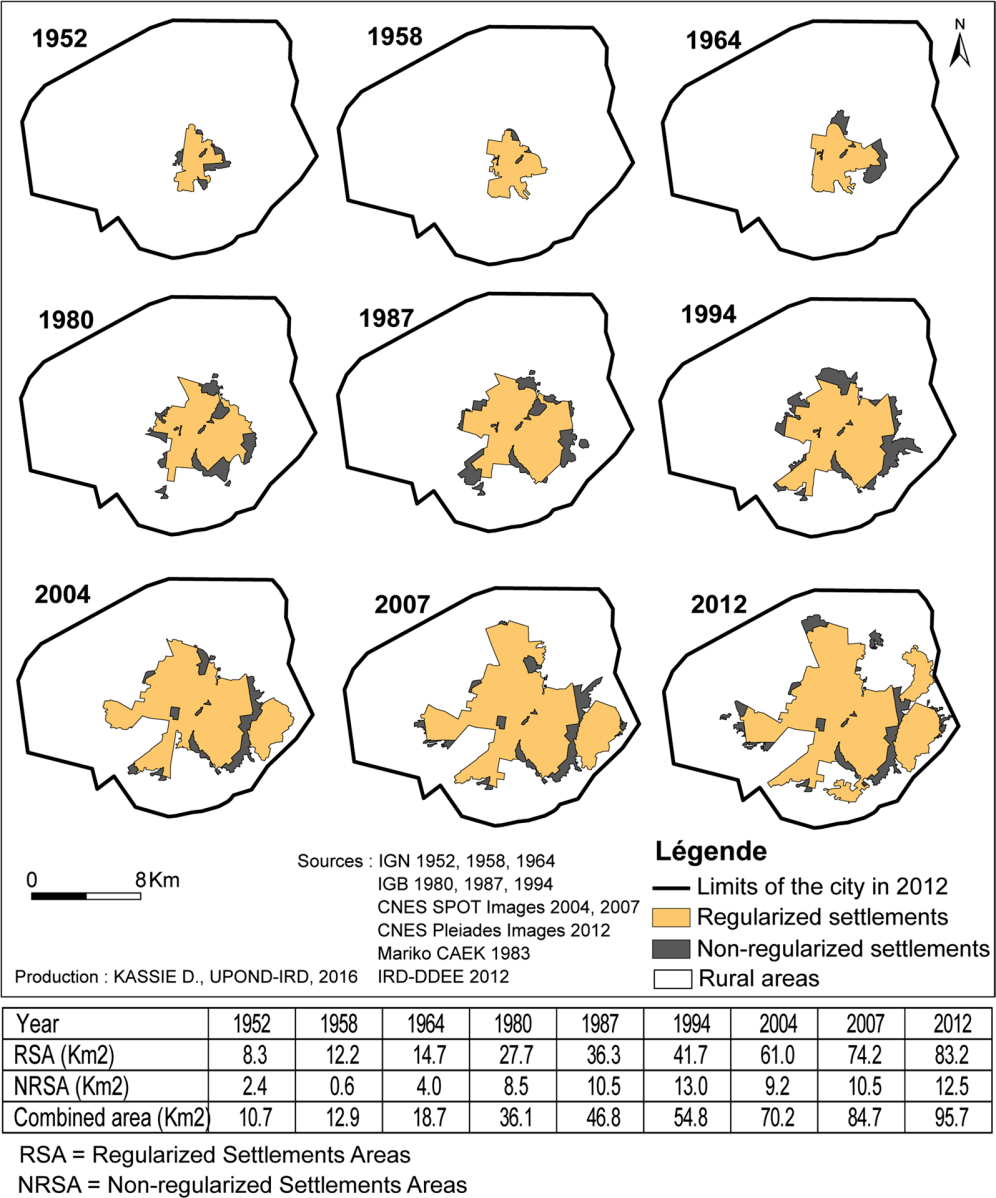
Expansion of Bobo-Dioulasso from 1952 to 2012

**Fig. 7:**
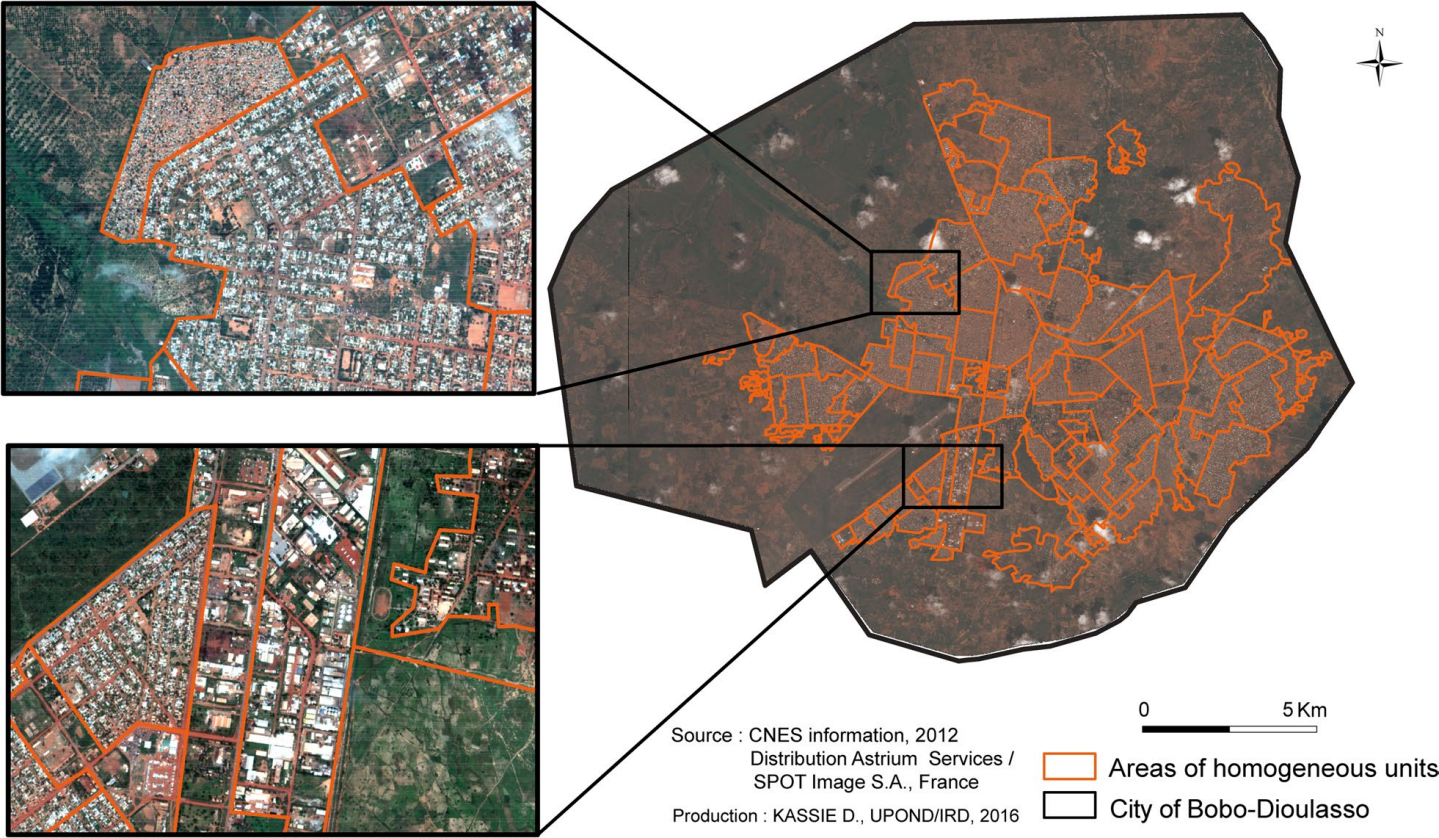
Division of Bobo-Dioulasso in 125 areas of homogeneous units (2012)

Between the beginning of its colonization and the independence, the population of the second city of Burkina Faso, which is presented as the economic capital, increased from 3000 to 50,000 inhabitants, to reach 230,000 inhabitants in 1975, 310,000 in 1996 and 490,000 in 2006 [27]. This growth led to a radial extension of the city without specific building densification due to the absence of physical constraints. According to the urban growth analysis based on aerial photographs and satellite images, the city surface increased from 10.7 km^2^ in 1952 to 95.7 km^2^ in 2012 (Fig. 6). Analysis of the urban morphology showed that Bobo-Dioulasso could be divided in 125 AHU that were well differentiated and that could be easily distinguished on the basis of the density of buildings, vegetation and roads (Fig. 7).

**Fig. 8:**
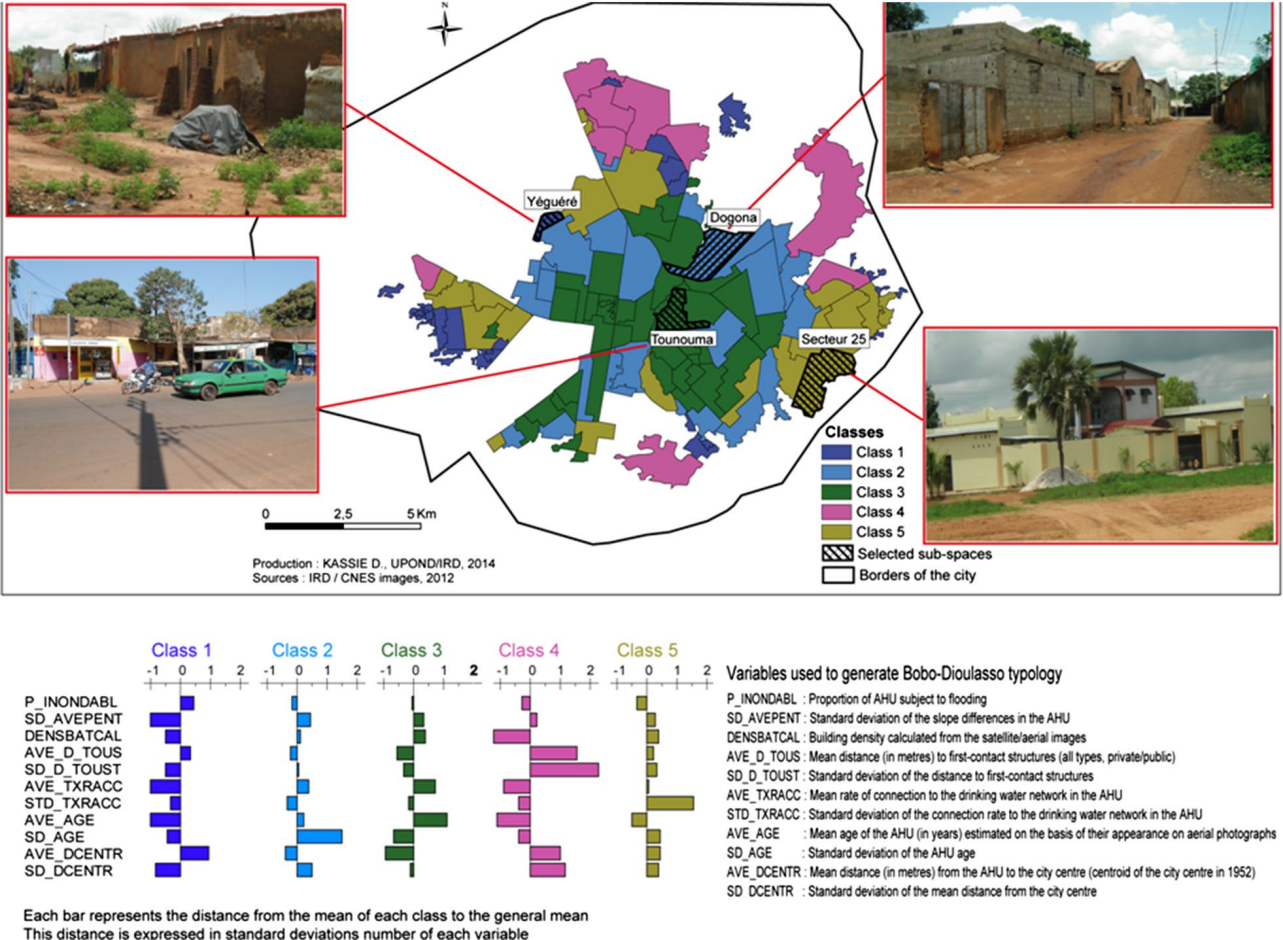
Spatial distribution of the AHU in five classes and location of the selected sub-spaces

The spatial analyses based on AHU allowed dividing the city in five classes that explained almost 50% of the total variance (Fig. 8). Class 1 (C01; in dark blue) corresponded to peripheral areas under development, but with few infrastructures (both regularized and non-regularized settlements). Class 2 (C02; in light blue) included areas of different age, sometimes distant from the city centre, but globally well-equipped in infrastructures. Class 3 (C03; in green) grouped together central areas that were urbanized long ago, densely built and well equipped. Class 4 (C04; in pink) corresponded to peripheral AHU under development and with low population density. Class 5 (C05; in olive green) included the recent peripheral areas under development and well equipped.

As there was not enough population to be included in the Class 4, it was not considered for the population survey. Finally, four AHU were randomly selected for the population survey to describe the health disparities: Yéguéré (CO1), Dogona (CO2), Tounouma (CO3) and Secteur 25 (CO5). They were characterized by very different conditions of urbanization, position within the city, time of creation and access to healthcare structures (Fig. 8 and Table 2).

**Table 2:** Features of the chosen sub-spaces

Sub-space	Age of the neighbourhood (years)	Parcelling by-law	Proximity to centre^a^ (m)	Proximity to health infrastructures (m)
Tounouma	>55	Yes	1000	750
Dogona	51	Yes	300	900
Secteur 25	17	Yes	600	900
Yéguéré	26	No	500	1000

^a^Barycentre of the town in 1952

Finally, 3400 eligible plots were selected by several spatial random sampling without replacement, among the 8812 plots identified from the Pléiades mosaic image (Table 3). A first random selection of 350 plots for each sub-space was carried out to offset the problems of uninhabited plots, wrong plot identification in the satellite images, absence of people, possible refusal and non-eligibility of households (single-member, non eligible children or adults). Additional random samplings were carried out after the removal of already visited plots to achieve the aim of 250 households by AHU.

**Table 3:** Distribution of plots in the sub-spaces under study

Sub-space	Plots on the Pléiades mosaic image	Inhabited plots	Randomly selected plots	Eligible plots
Dogona	2310	2124	700	262
Secteur 25	3281	2852	1400	290
Tounouma	600	600	600	298
Yéguéré	2621	2621	700	234
Total	8812	8197	3400	1084

**Fig. 9:**
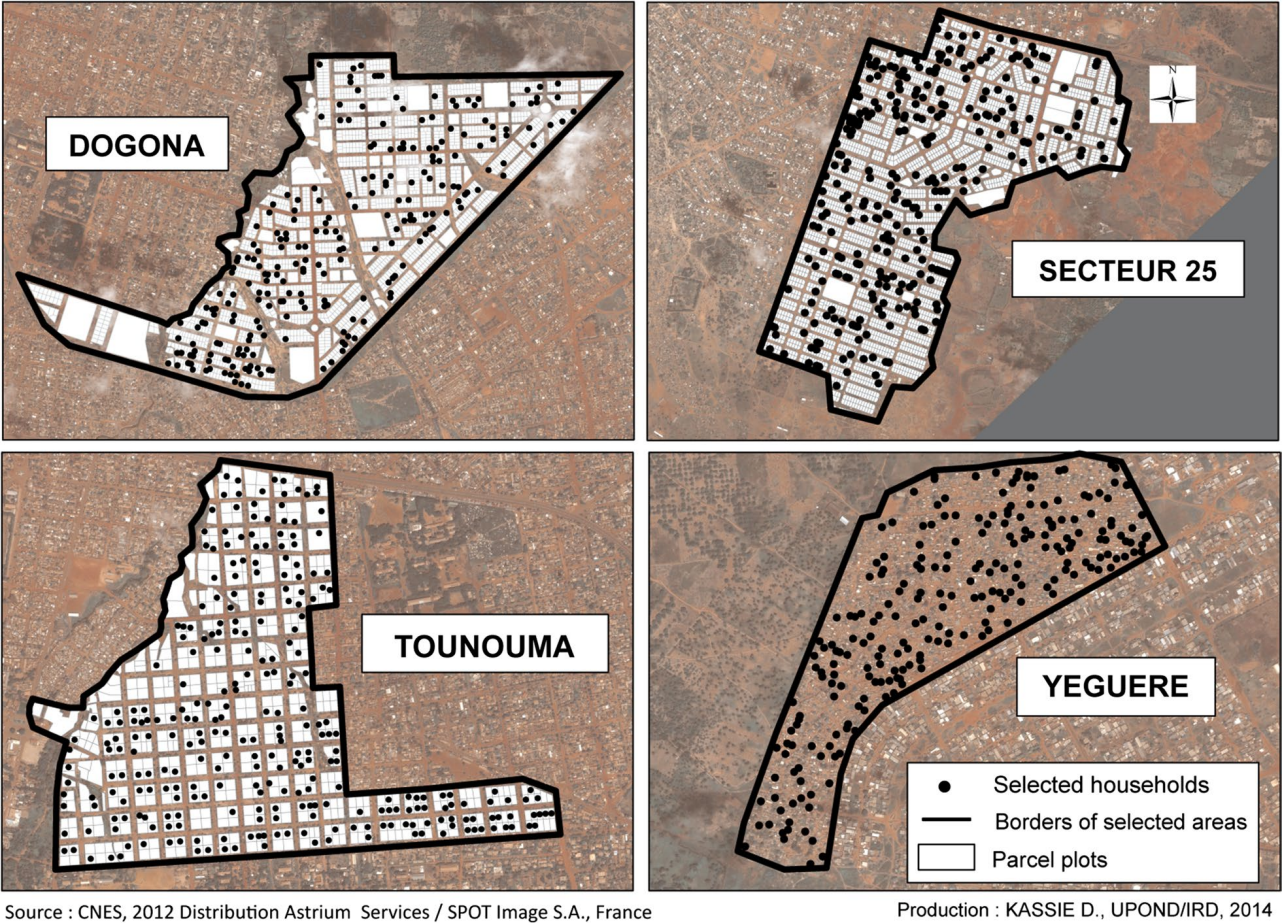
Spatial distribution of the randomly selected households for the survey

In the eligible plots (1084), 1320 households (1320/2192; 60.2%) were included in the survey. Finally, 1045 households were surveyed (Table 4 and Fig. 9).

**Table 4:** Distribution of the surveyed households in the different sub-spaces

Sub-space	Number of visited households	Number of eligible households	Number of surveyed households
Dogona	500	279	235
Secteur 25	490	329	290
Tounouma	964	479	286
Yéguéré	238	234	234
Total	2192	1320	1045

**Fig. 10:**
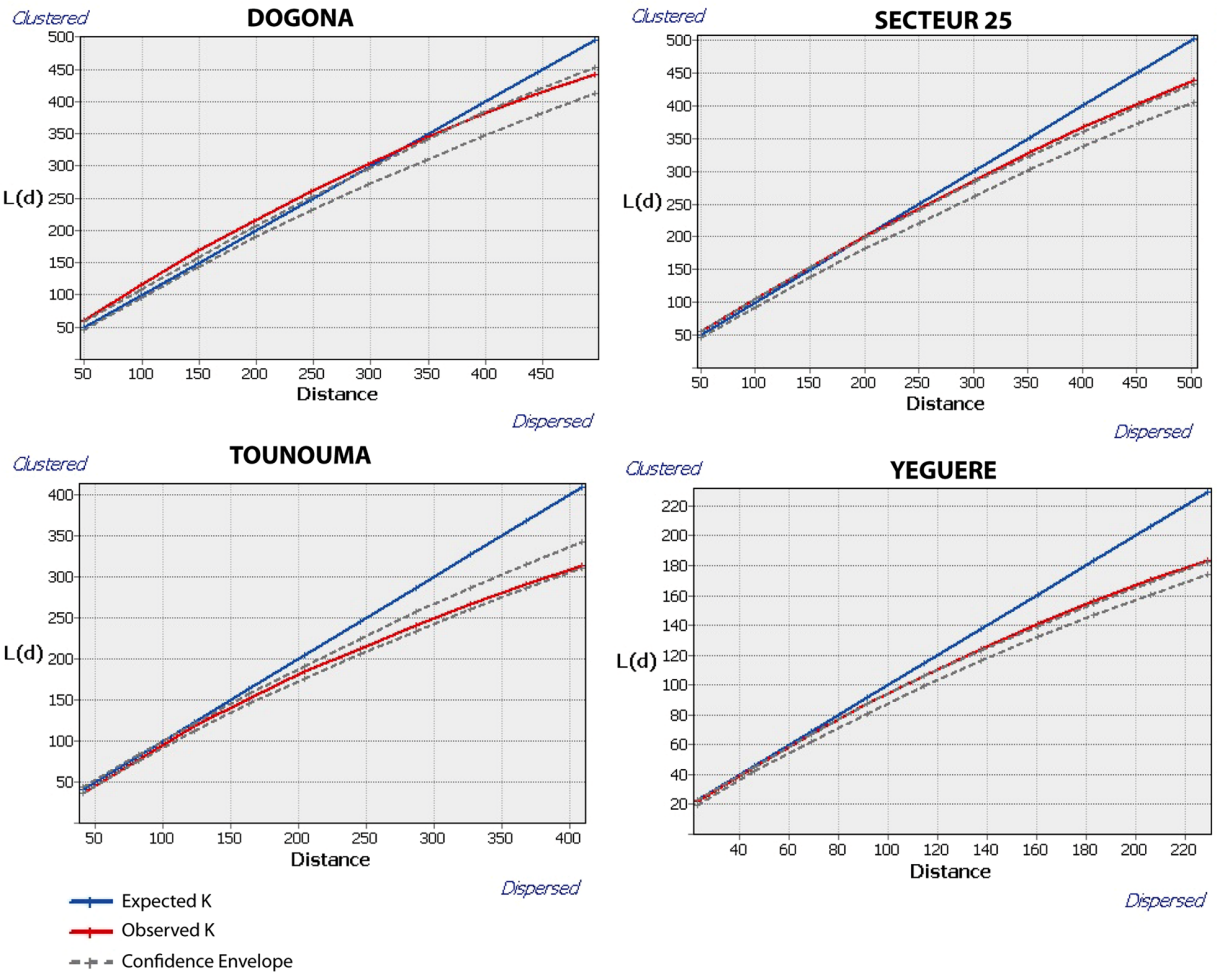
Analysis of the spatial distribution of the households surveyed in each district by the Ripley’s K function. When the observed K-value (in *red* on the graphic) is higher than the expected one (in *blue* on the graphic) for a particular distance, the household distribution is more clustered than the random distribution at the considered distance. When the observed K-value is lower than the expected one, the household distribution is more dispersed than the random distribution at the considered distance

The analysis of the spatial distribution of the surveyed households by Ripley’s K function showed that in Dogona, households were not randomly distributed. Within 300 m radius, the households appeared concentrated. The spatial structuring of the area which is divided by two rivers might explain this situation. In the other districts, the households could be considered as dispersed. In Dogona, analysis of the morbidity should take into account the spatial aggregation of households, while in the other districts a concentration of cases could not be related to the sampling (Fig. 10).

Finally, 860 adults (35–59 years of age) and 883 children (6–59 months of age) were surveyed (Table 5).

**Table 5:** Distribution of surveyed adults and children in the different sub-spaces

Sub-space	Men	Women	Total	Boys	Girls	Total
Dogona	84	109	193	103	99	202
Secteur 25	79	136	215	119	105	224
Tounouma	111	142	253	119	129	248
Yéguéré	104	95	199	113	96	209
Total	378	482	860	454	429	883

The number of surveyed men was lower than that of women, but the difference was not significant with respect to the data registered during the demographic and health survey carried out in Burkina Faso in 2010 (*p* = 0.25) [28].

## Discussion

The objective of the study carried out in Bobo-Dioulasso was to highlight health disparities linked to differential urbanization due to non-homogeneous urbanization processes. Therefore, this sampling method was developed with the aim of identifying sub-spaces as different as possible, starting from the hypothesis that the health status of populations living in different environments is different. Thus, five different types of urbanization could be characterized. They were then used to guide the approach for the analysis of the health status within Bobo-Dioulasso through the selection of four sub-spaces that are representative of the urban diversity. The results show that a multifactorial approach, which includes the use of spatial information on the urbanization process, the urban morphology and the access to infrastructures, allows meeting this objective [29].

Several studies have demonstrated the value of the spatial sampling methods for population health surveys in developing countries where little information is available [30–33].

In such approaches, surveyors must follow a standardized procedure to find the randomly generated points in the field and select the nearest household or group of households for surveying [13, 34]. In Bobo-Dioulasso, the households could be located at their exact geographic position and the surveyor had to move to another random point integrated in his GPS unit in case of ineligibility or refusal. The randomly points to survey may be generated within different methods. Escamilla et al. [33] used Google Earth imagery to digitalize household structures in a rural area and then to produce a random sample from the list of generated households. In our study, we rather used the plot centres (regular areas) or the house coordinates (irregular areas). Lowther et al. [30] applied a similar method for a health survey in an urban area in Zambia. They showed that this method offered an alternative sampling technique which allowed besides the reduction of the selection bias of the households. All these studies highlighted the accuracy of such approaches as well as their time and cost efficiency.

Our method showed its feasibility in regularized areas, such as the district of Tounouma, but also in non-regularized areas, such as the district of Yéguéré. It could be applied also in other cities. Moreover, the typology of Bobo-Dioulasso could be used in other studies, or even for the development of a system for demographic monitoring.

Although the techniques of multifactorial analysis and classification allow a good synthesis of the information and the distribution of the whole space in different classes, the question of the choice of the spatial unit to privilege within the identified classes remains essential. In Bobo-Dioulasso, the good knowledge of the area and the qualitative surveys carried out before these analyses could guide our choice. It should be also noted that the digitalization of all the houses of the non-regular sub-space is time consuming. In addition, this sampling frame may require a training for the use of GPS units and the reading of maps. In terms of tools and material, we must stress that the development of this sampling method required the acquisition of relatively expensive data (aerial photographs and especially satellite images), the use of expensive software programmes, such as ArcGis, and specific skills for the analyses. Some of these costs could be reduced by using open-source software, such as QGis, and data provided by the OpenStreetMap community and by using free-of-charge satellite images, for instance Sentinel-2 imagery.

## Conclusion

Different sampling methods can be used to carry out cross-sectional population surveys (i.e., stratified and non-stratified random, by purposeful sampling, cluster sampling). Overall, the aim of such methods is to obtain a good representativeness of the space or of the population that seems impossible to reach in the context of a city due to its complexity. Moreover, these methods cannot be always implemented without multiplying the bias, particularly in low-income countries, due to the lack of data availability and quality. More often they were aggregated at the scale of the whole city or of specific administrative zones (urban sectors, areas serviced by drinking water or waste collection) that were not always overlapping and that were not necessarily appropriate for studying health questions.

However, our approach shows that alternatives to sample an urban population in a low-income country exist. It was applied in Saint-Louis of Senegal as part of the SANTINELLES project in which this study fitted [35].

## Abbreviations

HAC: hierarchical ascending classification
AHU: area of homogeneous units
GIS: geographic information system
CNES: Centre National d’Etudes Spatiales
GPS: global positioning system
IGB: Institut Géographique du Burkina
IGN: Institut Géographique National
ONEA: Office National de l’Eau et de l’Assainissement
PCA: principal component analyses
SANTINELLES: SANTé, INégalités villES
UPOND: Université Paris Ouest Nanterre la Défense

## Footnotes

1 Simple random sampling is a type of probability sampling in which observations are selected randomly within a population with a known sampling probability or fraction. (http://documentation.statsoft.com/STATISTICAHelp.aspx?path=Glossary/GlossaryTwo/S/SimpleRandomSamplingSRS).

2 Data were analysed with the Envi (image analysis), ArcGIS (database and mapping) and Philcarto (statistical cartography) software programmes.
